# Draft Whole-Genome Sequences of *Salmonella enterica* subsp. *enterica* Serovars Enteritidis, Veneziana, and Salford, Isolated from Herbs

**DOI:** 10.1128/genomeA.00134-16

**Published:** 2016-03-24

**Authors:** Stephen Frink, Christina Morales, David Kiang

**Affiliations:** California Department of Public Health, Food and Drug Laboratory Branch, Richmond, California, USA

## Abstract

*Salmonella* is a foodborne pathogen found in a wide variety of sources. Here, we report draft genome sequences of three *Salmonella enterica* subsp. *enterica* serovars found in herbs: Enteritidis, Veneziana, and Salford, with the latter two being extremely rare in California.

## GENOME ANNOUNCEMENT

*Salmonella enterica* is a foodborne pathogen and a major cause of bacterial gastroenteritis in the United States and worldwide (1, 2). It can be found in a wide variety of animal and environment sources. *Salmonella* spp. have a high tolerance to desiccation and can survive on untreated dried herbs and spices (3). The consumption of these herbs and spices can serve as a source of human infection. The three genome sequences reported here were isolated from herbs. The first, *Salmonella enterica* subsp. *enterica* serovar Enteritidis (accession no. FDLB_F12M01377), was isolated from wormwood. The second and third serovars, *Salmonella enterica* subsp. *enterica* Veneziana (accession no. FDLB_F12M01383-1) and Salford (accession no. FDLB_F12M01383-2), were isolated from mullein leaf powder.

The *Salmonella* strains were isolated from herbs during a California Department of Public Health (CDPH) investigation using a modified Bacteriological Analytical Manual (BAM) method for culturing *Salmonella* (http://www.fda.gov/Food/FoodScienceResearch/LaboratoryMethods/ucm070149.htm). Genomic DNA was extracted using the DNeasy blood and tissue kit (Qiagen, Valencia, CA) on 18- to 24-h cultures. The genomes were sequenced on the Ion PGM (Life Technologies) using an Ion 316 Chip version 2. SPAdes (4) was used for *de novo* assembly, and all contigs <500 bp or with <2× coverage were removed. The resulting assemblies consisted of 42, 109, and 89 contigs for FDLB_F12M01377, FDLB_F12M01383-1, and FDLB_F12M01383-2, respectively. The total assembly size for FDLB_F12M01377 (*S*. Enteritidis) was 4.98Mbp, with 76.3× average coverage and *N*_50_ value of 391 kbp. For FDLB_F12M01383-1 (*S.* Veneziana), the total assembly size was 5.05 Mbp, with an average coverage of 84.4× and *N*_50_ value of 138 kbp. The total assembly size of FDLB_F12M01383-2 (*S.* Salford) was 4.82 Mbp, with an average coverage of 35.7× and *N*_50_ value of 156 kbp. The draft genomes were annotated using the NCBI Prokaryotic Genome Annotation Pipeline (http://www.ncbi.nlm.nih.gov/genomes/static/Pipeline.html). Plasmid sequences were identified using PlasmidFinder 1.2 (5).

Two plasmid sequences were identified in FDLB_F12M01377, one of them being a virulence plasmid containing the *svp* locus (6). It also contained intact *Salmonella* pathogenicity islands (SPI)-1, -2, -3, and -4, as well as a partially complete SPI-5. Interestingly, FDLB_F12M01377 lacked the *Salmonella* difference region I, a common marker for *S*. Enteritidis (7). FDLB_F12M01383-1 had one plasmid sequence and intact SPI-1, -2, -3, and -4. FDLB_F12M01383-2 lacked plasmid sequences but had SPI-1, SPI-2, and SPI-4. None of the plasmids from these isolates contained known drug resistance genes. The presence of virulence factors in these isolates suggests that *Salmonella* strains found in herbs have the potential for pathogenicity.

### Nucleotide sequence accession numbers.

The draft whole-genome sequences of FDLB_F12M01377, FDLB_F12M01383-1, and FDLB_F12M01383-2 have been deposited in GenBank under the accession numbers LFCD00000000, LFCE00000000, and LFCF00000000.
